# The effect of interventions on the transmission and spread of HIV in South Africa: a phylodynamic analysis

**DOI:** 10.1038/s41598-018-37749-3

**Published:** 2019-02-25

**Authors:** Eduan Wilkinson, Dennis Maletich Junqueira, Richard Lessells, Susan Engelbrecht, Gert van Zyl, Tulio de Oliveira, Marco Salemi

**Affiliations:** 1 Emerging Pathogens Institute, University of Florida, Gainesville, Florida 32608 United States of America; 2 KwaZulu-Natal Research Innovation and Sequencing Platform (KRISP), Nelson R Mandela School of Medicine, University of KwaZulu-Natal, Durban, 4001 South Africa; 3 School of Laboratory Medicine and Medical Science, Department of Health Sciences, University of KwaZulu-Natal, Durban, 4001 South Africa; 4 Division of Medical Virology, Department of Pathology, Faculty of Medicine and Health Sciences, Stellenbosch University, Tygerberg, Cape Town South Africa; 5 National Health Laboratory Services (NHLS), Tygerberg Coastal, Cape Town South Africa

## Abstract

The epidemic in South Africa is characterized by high genetic diversity driven by multiple independent introductions. The bulk of these introductions occurred between 1985–2000 during which time HIV prevalence increased exponentially. Epidemic growth has stabilized in recent years with the implementation of several interventions. Here we identified distinct HIV clades from a large sequence dataset of southern African HIV sequences (n = 15,332). Each clade was characterized using phylodynamic and phylogeographic methods to infer their growth through time and space. The estimated date of origin for the 18 clades that were found, fell between 1979–1992 with strong growth during the 1990’s. Phylogeographic reconstruction revealed wide dispersal of clades throughout the country with the city of Johannesburg as the focal point of viral dispersal. We found clear signs of decreasing growth rate in four of the clades since the advent of interventions, while other clades have continued to growth and expand. Our results demonstrate that interventions do not affect the HIV epidemic universally with major difference between different clades over time and space. Here we demonstrate the utility and flexibility of molecular epidemiological methods and demonstrate how they can potentially be a powerful tool in HIV epidemic monitoring in South Africa.

## Introduction

South Africa and southern Africa falls in the epicentre of the HIV/AIDS pandemic. The region is home to <2% of the world’s population, yet accounts for a third of the global HIV disease burden. The HIV epidemic in South Africa exploded in the 1990’s. Prevalence measured amongst women attending antenatal clinics increased from <1.0% in 1990 to ~24.5% by the end of the decade^1^. The South African government’s response to the epidemic was initially slow, but since the early 2000s, has become more responsive. The government’s reversal on their prior policy stance – that HIV does not cause AIDS – has been central to this change. Several national campaigns to combat the spread of the virus been launched such as prevention of mother to child transmission (pMTCT), as well as the combination antiretroviral therapy (cART) and medical male circumcision. In September of 2016, the government further committed to the adoption of the UNAIDS 90–90–90 strategy, which aims at having 90% of infected individuals diagnosed by 2020, 90% of whom should be on cART, and 90% of those on cART virologically suppressed^2^.

In the context of mass interventions to stop the transmissionim and spread of HIV such as cART, there is a growing need for tools or studies that can measure their effects on the epidemic. Standard epidemiological techniques would traditionally be the preferred choice of platform for such studies. However, in recent years, molecular epidemiology studies have emerged, which can provide a broad nuanced view of the epidemic. This makes molecular epidemiology studies attractive measures to complement traditional epidemiological studies^3–5^. These studies draw from recent advancements in sequencing technologies, computational biology and the large amount of HIV sequence data routinely being generated as part of standard of care (i.e. drug resistance testing). The added advantage of molecular epidemiology studies over traditional methods is that they are more cost effective and quicker to perform.

Previously, we reconstructed the history of the HIV subtype C epidemic in South Africa to shed light on when the epidemic emerged, as well as to dynamically model changes in the past viral population size of the epidemic. Molecular clock analysis placed the time to the most recent common ancestor (tMRCA) of the epidemic around 1960 with the 95% confidence intervals (CI) ranging between 1956 and 1964^6^. More over, a random longitudinal sampling of viral linages within each southern African country will produce similar estimated dates for subtype C. Therefore, its clear that the southern African HIV-1 subtype C epidemic share a common ancestor around the 1960. Phylodynamic modelling also revealed periods of strong epidemic growth during the late 1970 s and throughout the 1980s in the southern African region. However, when analysing only South African sequences the period of epidemic growth appears to be approximately ten years later with most growth in the mid-80’s and throughout the 1990’s. This 10-year lag in the epidemic growth phase of the South African epidemic is also consistent with HIV prevalence estimates. By the start of the 1990 s the HIV prevalence rate in South Africa was ~1.0% of the adult population, while prevalence in Zimbabwe at the time was ~10.0%^7^. In phylogenetic reconstructions of the southern African epidemic we also observe a strong panmixia pattern of dispersal of southern African sequences – with no clear spatial clustering (i.e. lack of supported monophyletic clades including strains specific geographic area).

Based on such findings, we hypothesized that several independent introductions of HIV subtype C into South Africa over time must have occurred to give rise to the viral genetic heterogeneity we observe today. To this end, we conducted a phylogeographic study of a large southern African dataset (n = 11,289) to infer the number of viral exchanges (imports and exports) of HIV between South Africa and its neighbours through a phylogeographic reconstruction^8^. The results confirmed our hypothesis and identified that the bulk of viral exchanges occurred between 1985 and 2000 with little to no subsequent events. This period of viral introductions coincided with mass inward migration of people from neighbouring countries into South Africa as Apartheid was abolished and South Africa was re-integrated into the Southern African Development Community (SADC). From these results, it is clear that the HIV epidemic in South Africa is actually a composite of multiple, parallel sub-epidemics spreading in the country at the same time, giving rise to the heterogeneity we observe today. This implies that even though the subtype C epidemic in South Africa share a common ancestor as far back as 1960, it does not mean that HIV was already circulating in the country at the time. Rather, a large number of independent introductions from across the southern African region much later (1985–2000) has shaped the epidemic, giving rise to the extreme genetic diversity we observe today.

We used a similar approach as that used by Novitsky and colleagues^3^, in the present study. We analysed a large sequence dataset of HIV patients from South Africa and its neighbouring countries to identify clades that represent sub-epidemics. We applied phylodynamic and phylogeographic methods to each clade to infer their temporal origin and growth as well as their spatiotemporal distribution. We were particularly interested in two main questions: (*i*) What temporal changes in the growth of clades have occurred, particularly in the period of interventions (2005–2015); and (*ii*) What has been the spatio-temporal characteristics of clades?

## Results

### Dataset and clade identification

Aggregated viral genotypes from four different sources produced a sequence dataset of 15,332 sequences. Subtyping and recombination analyses identified 75 sequences that were not pure subtype C isolates or showed statistically significant signs of viral recombination. These 75 sequences were removed from the sequence dataset, which resulted in a final dataset of 15,257 sequences. No duplicate sequences were identified. Manual editing produced a codon alignment of 1,088 base pairs (bp). The final length of the alignment after 33 codon positions associated with major drug resistance mutation sites were removed was 989 bp long. A breakdown of the 15,257 subtype C *polymerase* (*pol*) sequences is provided in Supplementary Figure 1.

**Figure 1:**
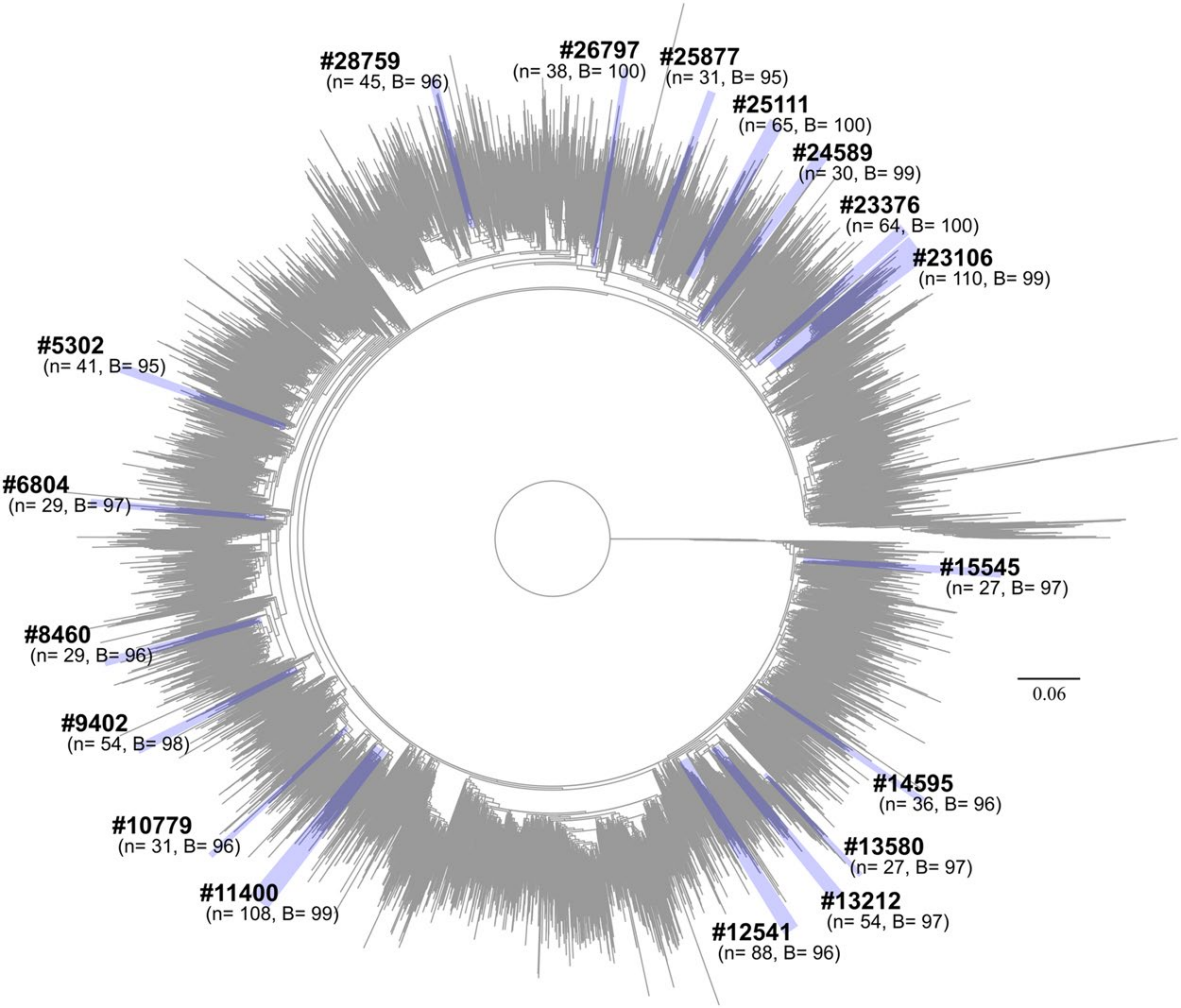
Large-scale phylogenetic reconstruction of the HIV-1 subtype C epidemic in the southern African region. The tree contains 15,257 HIV subtype C pol sequences from across the southern African region and is rooted with the HXB2 reference strain. The maximum likelihood phylogeny was construct in IQ-TREE (GTR + I + G) with 1,000 bootstrap replicates. The scale bars at the bottom represent the number of substitutions per site along branches in the tree topologies. The 18 clades we identified in the ML-tree topology at an 8% genetic distance threshold in PhyloType are highlighted in blue and annotated according to the PhyloType IDs as in Table 1. The values in brackets below each sequence ID represent the number of sequences in that clade (*n*), while the last number (*B*) represents the bootstrap support for the parental branch for each clade.

We used the 15,257 subtype C sequences to construct a maximum likelihood (ML) tree topology that was used to identify clades in PhyloType. We first conducted a sensitivity analysis in PhyloType increasing the genetic distance threshold by 0.5% incrementally starting at 2% and working up towards 10%. Based on this exercise, an 8% genetic distance cut-off was identified as optimal. At this threshold, we identified a total of 18 clades of interest containing 907 South African sequences (Fig. 1). The largest contained 110 sequences that were sampled over the course of ~15 years, while the smallest contained 27 sequences that were sampled over the course of ~9 years (Table 1). Of the 18 clades, 14 had good temporal signal as determined by TempEst, which would allow for phylodynamic and phylogeographic reconstruction. The remaining four had a negative correlation between genetic diversity and sampling times and was not suited for molecular clock analyses.

**Table 1:** Summary of the 18 clades that were identified in the PhyloType analysis of the ML-tree.

Clades	R^2^ from TempEst	Date range (in years)	Size of clade	Intra-cluster genetic diversity (%)	Bootstrap support (%)
#5302	0.213	16.753	41	6.50	95
#6804	0.355	18.460	29	6.90	97
#8460	0.086	9.847	29	5.40	96
#9402	0.329	8.838	54	6.00	98
#10779	0.137	8.710	31	6.50	96
#11400	0.172	11.279	108	6.70	99
#12541	0.116	17.581	88	7.40	96
#13212	0.219	17.471	54	7.10	97
#13580	0.154	8.288	27	5.60	97
#14595	0.233	8.460	36	7.50	96
#15545	0.069	9.299	27	6.30	97
#23106	0.217	15.134	110	7.80	96
#23376	0.287	7.784	64	7.00	100
#24589	0.285	10.819	30	6.80	99
#25111	0.033	11.460	65	7.20	100
#25877	0.326	8.825	31	7.90	95
#26797	0.056	8.647	38	8.00	100
#28759	0.159	10.310	45	7.80	96

### Phylodynamic characterization of clades

**Figure 2:**
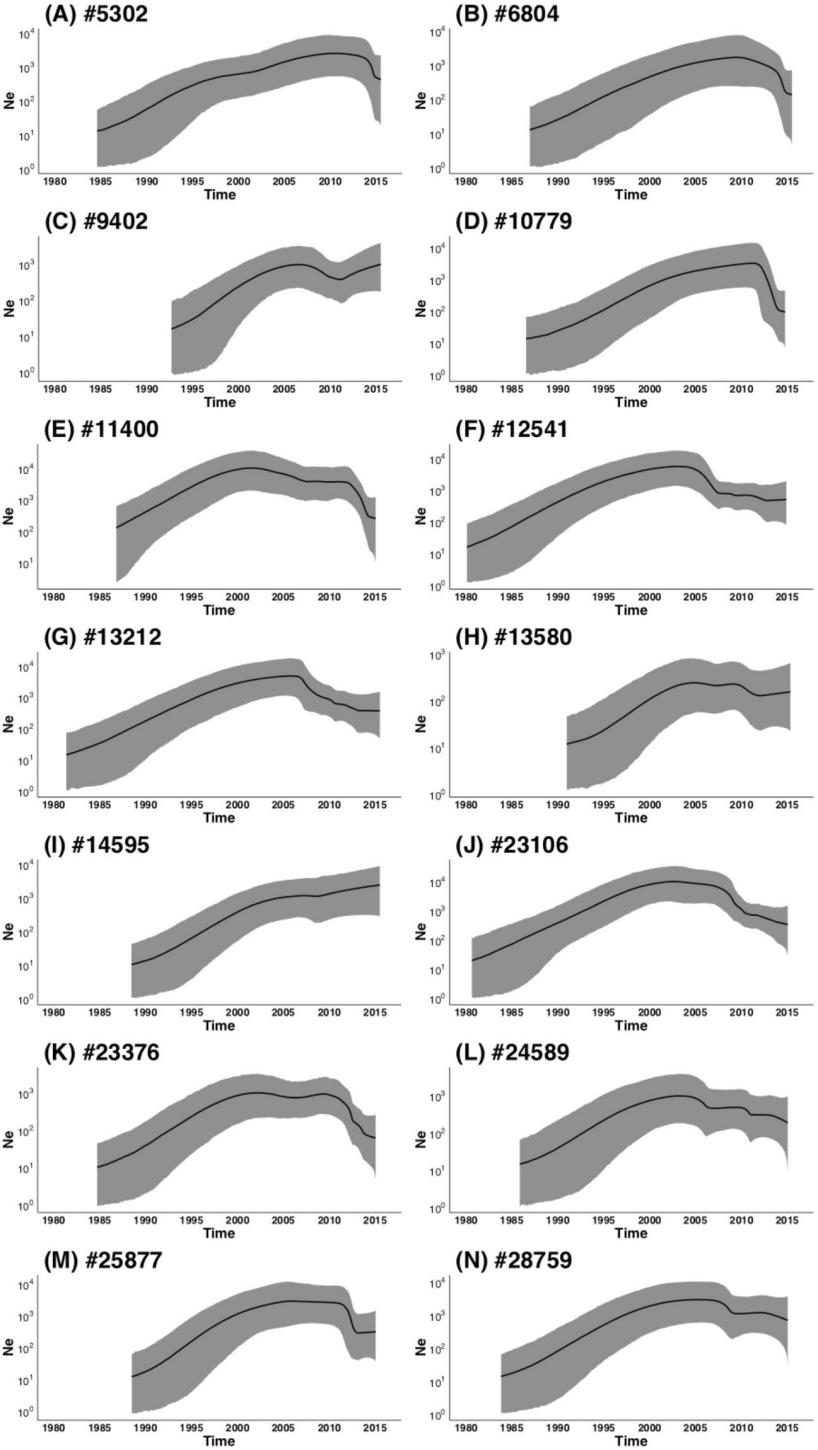
The inferred phylodynamic trends of 14 South African clades. On the x-axis time in calendar years are represented while on the y-axis the effective population size (*Ne*) are presented. These phylodynamic plots were inferred in Tracer using the mean tree height estimate. The grey shaded area represents the 95% confidence interval for Ne estimates, while the black line represent the median estimate through time.

The 14 transmission clades that had good temporal signal were analysed under various coalescent tree priors in a molecular clock framework to infer their estimated dates of origin (Supplementary Figure 2) as well as their past population growth dynamics (Fig. 2). Using the marginal likelihoods inferred under the different coalescent tree priors, Bayes factor calculations favoured the use of a SkyRide coalescent tree prior over the use of all other models. This was the case for all of the clades. Based on these SkyRide estimates the inferred tMRCA for clades fell in the period between 1979 and 1992. The oldest clade (#12541) had an estimated date of origin around 1979.5 (95% CI: 1973.5–1985.4) while the youngest clade (#9402) had an estimated date of origin around 1992.2 (95% CI: 1987.1–1996.7). These estimated dates of origin are consistent with our previous findings that showed many viral introductions into South Africa from neighbouring countries during the period^8^.

Phylodynamic modelling of the past population growth through time of the 14 clades revealed strong exponential growth in the effective population size (*Ne*) throughout the 1980’s and 90’s. Since 2000 the growth in clades appears to have stabilized. These observations are in concordance with the rising prevalence trends of HIV that was observed in South Africa during this period. We observed decreases in the effective population size for several clades (#5302, #6804, #10779, #11400, #12541, #13212, #23106, #23376 and #25877) in more recent years. These decreases coincide with the period of interventions (2005–2015), such as the role-out of cART and medical male circumcision campaigns. However, these decrease were not observed in all of the clades. For example, in clade #9402, a small period of decline in the population size was observed between 2005 and 2010, but has since entered a new growth phase. Similarly, small decreases was observed for clades #13580, #24589 and #28759 however these decreases were not significant based on their 95% confidence intervals. Finally, instead of a decrease, a small increase in growth was observed for clade #14595 over the period from 2010 to 2015.

### Birth-Death coalescent reconstruction of epidemiological parameters

**Figure 3:**
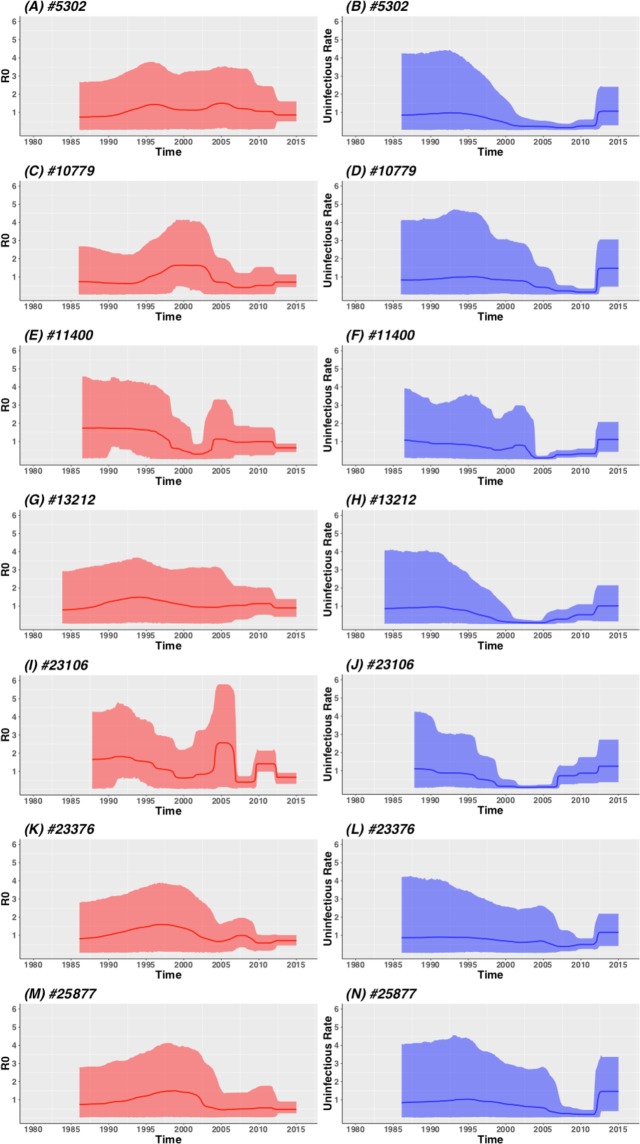
The inferred temporal changes in the estimated basic reproductive number and becoming uninfectious rate for seven South African clades for whom decreases in *Ne* was observed. The red solid lines represents the median estimate for R0 while the red shaded areas represent the 95% confidence interval for R0 estimates. The solid blue lines represent the median estimate for δ, while the blue shaded areas represents the 95% confidence interval for δ estimates.

Epidemiological parameters that were inferred from clades under the Birth-Death coalescent model (Fig. 3 and Supplementary Figure 3) correlates with our phylodynamic reconstruction of the past population size (*Ne*) of clades (Fig. 2). In four clades we observed the median R0 estimate as well as the 95% CI fall below 1 at the most recent time of sampling. The inferred temporal changes in R0 for clade #11400 revealed high R0 estimates for the period leading up to 2012 when R0 decreased to ~0.651 (95% CI: 0.420–0.887). On the other hand, the becoming uninfectious rate for this clade were very low leading up to 2012, when it increased to approximately a rate of 1.118 (95% CI: 0.432–2.081). For clade #23106, the inferred temporal changes in R0 suggest high growth potential up until 2006/2007 after which is decrease slightly for about two years. Our inference further suggests that this small decrease were followed by a small expansion (2010–2012) after which R0 decreased to a median of 0.676 (95% CI: 0.317–0.922). Conversely, the δ rate for this clade remains very low up until 2006/2007, which is followed by three incremental increases. At the last point of sampling the median δ rate was 1.245 (95% CI: 0.369–2.710).

The inferred temporal changes in R0 and the δ rate for clade #23376 revealed consistently high R0 estimates up until ~2009 after which R0 decreased. Conversely, the δ rate for this clade remained fairly low until ~2012 after which it increased. At the last time point of sampling the median R0 estimate was 0.708 (95% CI: 0.428–0.988), while the median δ estimated was 1.164 (95% CI: 0.409–2.188). Finally, our inferred temporal changes in R0 and the δ rate for clade #25877 revealed high R0 estimates leading up to 2005, after which it has decreased until ~2012. However, during this period the R0 95% CI remain fairly wide until ~2012 after which R0 decreased to a median of 0.475 (95% CI: 0.263–0.908). On the other hand, the δ rate for this clade remains very low throughout its lifespan up until ~2012, after which it increases to ~1.465 (95% CI: 0.382–3.365).

For the other clades for which we observed a decrease in the *Ne* (clades #5302, #6804, #10779, #12541 and #13212) at the most recent time of sampling, we observed decreases in R0 below 1, while the δ rate increases. However, the 95% CI for R0 at the most recent time of sampling still falls above 1, therefore we cannot be absolutely sure about the decreasing trend in these clades. For the remaining clades (#9402, #13580, #14595, #24589, #28759) the relatively high R0 estimates and low δ rates partially explains why some of these clades continue to grow and why some clades remain stable in the era of HIV interventions.

### Phylogeographic reconstruction of South African clades

**Figure 4:**
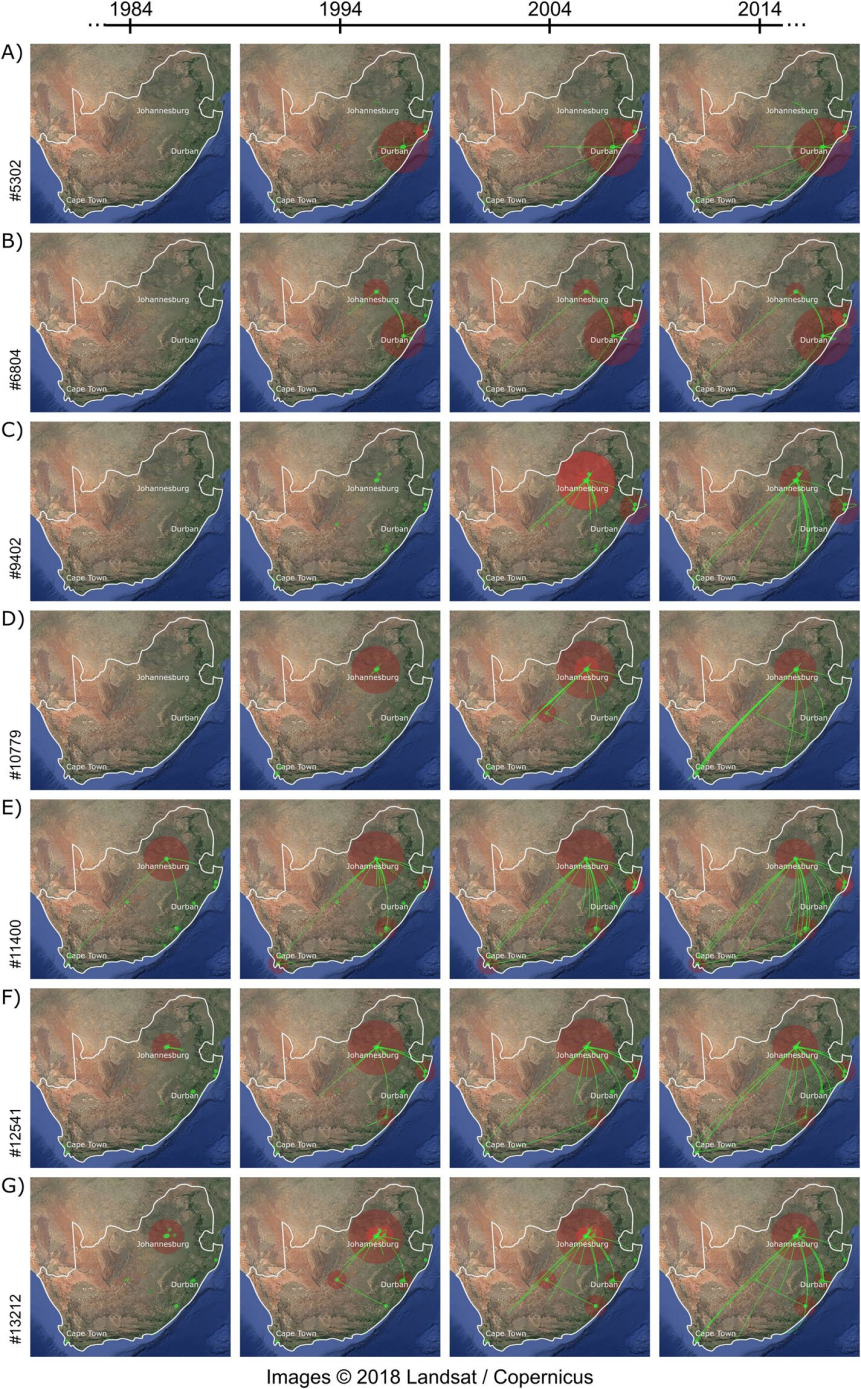
Temporal dynamics of HIV-1 subtype C spatial diffusion in seven South African clades. Lines between sites represent possible transitions of the virus between South African towns or cities. All transitions between locations have been plotted in this figure. Circle diameters are proportional to the square root of the number of MCC branches that maintain the same location state at each time-point. The map is based on satellite pictures available in Google Earth (http://earth.google.com).

We were able to reconstruct the geographic diffusion of transmission chains for 13 of the 14 clades with good molecular clock signal, which revealed a wide geographic circulation throughout the country. Keeping with prior convention, the phylogeographic reconstruction of the seven clades with clear signs of a decrease in the growth of clades are presented in the main text here (Fig. 4), while the remaining seven clades’ the phylogeographic reconstruction are presented in supplementary materials (Supplementary Figure 4). For clades #5302 and #6804, our phylogeographic reconstruction revealed the central region of KwaZulu-Natal as the most likely ancestral location for these transmission clades. For both clades our reconstruction inferred spread from this central location in KwaZulu-Natal to the northern regions of the province close to the Mozambican and Swaziland borders as well as viral spread to the city of Johannesburg by 2004. Since then these transmission chains have widely been dispersed including locations in Cape Town, Port Elizabeth, Mthatha and the central interior of the country.

The spatiotemporal reconstruction of all other clades revealed the city of Johannesburg, as the most like ancestral location for all of the other clades. From Johannesburg, clades appears to spread quickly to other locations within South Africa, including hyper endemic areas of KwaZulu-Natal, to Kimberly and Bloemfontein in the interior of the country, to Mpumalanga in the lowveld, and areas in the Western and Eastern Cape. Due to a lack of geographic variation in the sampling locations of sequences, phylogeographic reconstruction of clade #23376 could not be performed as 98% of this clade’s sequences were generated from sampling of HIV infected patients from Chris Hani Baragwanath Hospital in Soweto.

## Discussion

The phylogenetic reconstruction and phylotype analysis of the southern African HIV-1 subtype C epidemic revealed 18 South African clades of interest containing 907 sequences (9.7% of all South African sequences). Each one of these clades represents putative chains of HIV transmission within the country. Phylodynamic modelling of clades identified distinct growth patterns through time. Since the introduction and expansion of cART and other HIV interventions, the majority of clades decreased in size. However, in the context of these interventions others have actually expanded, which suggests that interventions do not affect the growth of clusters uniformly. More importantly, we have demonstrated here that molecular epidemiological inference of HIV can be used as a potentially powerful tool in the future to monitor the epidemic in South Africa. In the context of mass interventions such as cART and 90-90-90, these methods in the future has the potential to identify demographic groups or geographic areas where interventions are not performing optimally, complimenting existing surveillance of the epidemic. In turn the resulting information that is gained can be used to strengthen the public health response and design better targeted interventions amongst key demographic group or geographic areas.

Due to the high degree of genetic diversity that characterizes the HIV epidemic in the southern African region (Supplementary Figure 1D), we deliberately used very stringent criteria towards the identification of clades. Traditionally, closely associated viral strains of HIV are normally identified through the combined use of branch support and the use of a genetic distance threshold^3–5^. For the identification of transmission clusters, conservative cut-off values are normally used. For example, high branch support (>90%) and a low intra-cluster genetic distance threshold (2–4.5%)^4,5,9^. Our aim was not to identify highly similar clusters that represent putative chains of transmission, but rather to identify clades of HIV-1 subtype C in South Africa representing “sub-epidemics” resulting from independent introductions into the country. To this end we used a very conservative >95% branch support threshold cut-off. The use of a genetic distance threshold in molecular epidemiology studies, as with branch support thresholds, has often been an ad-hoc choice, with no clear cut-off, which prompted us to explore different genetic distance thresholds (Supplementary Figure 5). Our sensitivity analysis favoured the use of an 8.0% genetic distance cut-off to optimize the size and numbers of clades. In a recent publication we demonstrated that as you move from the terminal tips of the tree towards the root we observe a sharp decrease in Felsenstein’s bootstrap^10^. Therefore, given the relative depth of branches leading to our clades of interest and the large number of taxa in our phylogeny we are confident in the assignment of these clades.

Of the 18 clades that were identified, only 14 showed good signs of having good molecular clock signal. Bayesian coalescent reconstruction of the 14 clades place their estimated dates of origin in the 1980’s or early 1990’s. This falls within the 1985–2000 time period, which is consistent with our earlier findings, which proofed that several viral introductions into South Africa occurred during this time period that gave rise to several sub-epidemics. The reconstruction of the clades past population dynamics revealed strong growth in the *Ne* throughout the 1990’s (Fig. 3). These trends are consistent with HIV prevalence estimates over this time period as national prevalence trends increased from <1.0% to ~24.5%^7^. The reduction in the growth of *Ne* for some clades follows the initiation of the national cART campaign in 2004 and coincides with the expansion of access to therapy since 2009 and other interventions. In particular the decline and growth phase we observe in cluster #9402 really emphasises the potential power of molecular epidemiology methods to alert healthcare works of on going growth in some sup-epidemics, which can be used to design better targeted interventions.

However, the effective population size as a measure of relative genetic diversity only provides us with a nascent view of the past population dynamics. To investigate the observed decline in the *Ne* since the introduction of cART in greater detail we reconstructed the basic reproductive number of clades through time using a structured birth-death coalescent model^11^. The basic reproductive number provides an estimate of the number of new infections every infection would cause over the course of their infectious life. A R0 of one would indicate a stable epidemic with no growth or decline, while a R0 below one represents a shrinking epidemic (i.e. a decline in growth). Our birth-death coalescent reconstruction of the clades identified four (#11400, #23106, #23376 & #25877) where the median R0 estimate, including the 95% CI, were below one at the most recent time of sampling. This reduction in R0 was also accompanied with an increase in the uninfectious rate over the same time period. However, this decrease has only been observed in four of the fourteen clades on which molecular clock and birth-death coalescent modelling was performed. In the remaining clades no clear signs of decline in the R0 were noticed. It is therefore clear that some clades have stopped growing in the era of interventions (2005-present), while others have either remained stable or has continued to grow and spread. Given the lack of additional patient information that can be analysed in conjunction with the sequences it is difficult to ascertain exactly why other clades have continued to expand despite interventions such as increasing access to HIV testing and treatment.

The national cART campaign was launched in 2004. However, role out was initially slow with therapy only being administered at a CD4 cell count of <200-cells/ml blood^12^. Between 2004 and 2009, only ~600,000 people were initiated on therapy at this threshold. By 2009, government adjusted initiation criteria upwards to <350-cells/ml of blood for patients co-infected with HIV and TB and for all pregnant women who are infected. By 2013, initiation criteria were adjusted to <350-cells/ml blood for all patients and in 2015 was adjusted upwards again to <500-cells/ml blood. By 2015, ~3 million HIV positive South Africans were enrolled on therapy, making the South African national HIV treatment campaign the largest in the world, which constitutes a 30-fold increase in the number of individuals on cART when compared to 2005^12,13^ (Table 2). Since the introduction of therapy the national HIV incidence estimate amongst the sexually active population (aged 15–49 years) has decreased from 1.88 to 0.91. Of course its difficult to quantify what effect access to therapy has had, but it is safe to assume that this decline in incidence can in part be attributed to it. Conversely, the HIV prevalence rate amongst the same population group has remained stable over the same time period (2004–2017)^13^. This is to be expected as more HIV infected individuals are living longer due to the benefits of therapy. However, as of 2015 only 48.6% (95% CI: 46.0–51.2%) of HIV positive individuals were on cART. Additionally, of the HIV positive population in South Africa only ~26% were on cART and virally suppressed. This figure is extremely low considering that viral suppression is the most important determinant of future HIV incidence trends in the South Africa^12,14^.

**Table 2:** Number of HIV positive individuals on combination antiretroviral therapy (cART) since the start of the national treatment campaign in 2004.

Province	2004	2005	2006	2007	2008	2009	2010	2011	2012	2013	2014	2015
Eastern Cape (EC)	5200	11600	22000	38000	61000	92000	132000	186000	237000	287000	327000	359000
Free State (FS)	2300	4000	7000	14000	25000	42000	64000	91000	119000	147000	169000	187000
Gauteng (GT)	12400	29200	54000	95000	14000	208000	292000	409000	521000	631000	712000	774000
KwaZulu-Natal (KZ)	13500	27500	54000	106000	173000	262000	376000	521000	665000	807000	933000	1045000
Limpopo (LP)	2100	4400	9000	19000	34000	54000	81000	117000	148000	176000	198000	216000
Mpumalanga (MP)	3300	5800	11000	23000	37000	57000	84000	121000	165000	217000	267000	316000
Northern Cape (NC)	400	1200	3000	6000	9000	12000	15000	19000	24000	31000	38000	46000
North West (NW)	3000	7800	16000	28000	46000	63000	89000	122000	149000	173000	191000	204000
Western Cape (WC)	2500	10100	20000	32000	47000	65000	85000	108000	131000	154000	173000	190000
**Total**	**44700**	**98000**	**196000**	**361000**	**446000**	**855000**	**1218000**	**1694000**	**2159000**	**2623000**	**3008000**	**3337000**

A breakdown of the number of patients accessing treatment per province is provided while the total at the bottom represents the estimated number of HIV infected people on treatment nationally.

The phylogeographic reconstruction of transmission chains revealed a diverse geographic diffusion throughout the country over time (Fig. 3). The spatio-temporal reconstruction revealed linkage between urban and rural population centres across the country. Johannesburg appears to be a major centre for HIV transmission in South Africa. Phylogeographic reconstruction placed the estimated ancestral location of all but two of the clades in the city, with clades (#5302 & #6804) originating in KwaZulu-Natal from where they spread to Johannesburg and further throughout the country. The cluster for which phylogeographic reconstruction was not possible, due to constrains in its sequence locations, were predominantly sampled in and around the city of Johannesburg. The general trends we observed in our reconstruction supports the hypothesis that migrants, primarily in large urban areas, introduced HIV into South Africa from where it then spread onwards to rural communities. These urban-rural viral diffusion pathways coupled with high rates of infection has resulted in the extremely high prevalence rates that we now observe in hyper-endemic rural settings of KwaZulu-Natal such as those covered by the AHRI and CAPRISA population surveillance platforms^9,15^. These rural-urban transmission pathways and the vast geographical dispersal of clusters underscore the need for increased testing and treatment of individuals in high incidence and prevalence communities and the engagement of high-risk individuals (MSM, commercial sex workers, migrants and long distance truck drivers) throughout the country.

There are a couple of caveats to our reconstruction of the South African HIV epidemic. Firstly, our reconstruction was restricted to the use of a small fragment of the polymerase gene of the virus. The use of a small fragment from a single gene fundamentally limits our ability to accurately reconstruct the epidemic in the region and such a study would greatly benefit from the use of whole genome HIV sequences, as their use would assure better support for the branching structure in viral genealogies. However, the availability of whole genome HIV sequences from the region is limited to a small number contained in sequence databases (n < 500) or to very specific studies and research sites. The use of whole genome sequences for this study would therefore have restricted us to only a few hundred sequences as compared to several thousand when using *pol*. Furthermore, it has been demonstrated previously that HIV *pol* carries enough phylogenetic signal to accurately reconstruct phylogenetic clades/clusters^16^. Our study, using *pol* sequences, should therefore be able to reconstruct the underlying epidemic dynamics of HIV in the region to a reasonably good degree of success.

Secondly, given the convenient nature of sampling sources such as those drawn from drug resistance cohorts and public databases, it is evident that sampling from some geographic regions of South Africa and the southern African region might be underrepresented. For example, no sequences are currently available from Lesotho or Namibia, even though these countries are ranked second and fifth in the world in terms of HIV prevalence respectively. Their absence from any phylogenetic reconstruction of the epidemic in the region therefore constitutes a serious limitation. Moreover, within the South African context, sequences from North West province are lacking entirely, while genotypes from Mpumalanga, Limpopo, the Northern Cape and the Free State were generally underrepresented in comparison to the other provinces (Supplementary Figure 1). Conversely, the inclusion of sequences coupled to large demographic study sites such as ARHI and CAPRISA could lead to the overrepresentation of sampling from two hyper endemic settings in KwaZulu-Natal. The over- and underrepresentation of sequences from different geographic loci could therefore bias the phylogenetic and phylogeographic reconstruction that we were performed in the current study. Therefore, we can only reliably discuss viral spatial diffusion patterns between the four most highly sampled locations within the country (i.e. Gauteng, KwaZulu-Natal and the Eastern and Western Cape province). That being said, the results overwhelmingly support the hypothesis that Johannesburg was indeed the focal point of viral spread in South Africa, from where clades quickly spread to other urban centres and rural areas.

Finally, the genotype dataset that we used provided us with no other additional clinical, demographic, behavioural or socio-economic data. This has limited our reconstruction of the epidemic only to both time (temporal changes) and space (geographic spread). The inclusion of additional variables would have allowed us to identify how different risk factors contribute to HIV epidemic spread in the region. Such information would have been useful to determine why some clusters have shrunk since the introduction of cART and why some continue to grow and expanded in the presence of HIV interventions. This underscore the need for a national HIV sequence and patient database which could serve as the basis for the development of dynamic systems that can track the spread of HIV in the era of mass interventions.

In conclusion, our results clearly demonstrate that in the current context, where ~50% of HIV infected individuals are accessing treatment we could only observe clear decreases in the size of four of the clades we identified. Furthermore, our phylogeographic reconstruction underscores the wide geographic dispersal of HIV within South Africa. Given the wide geographic dispersal and the large scope of the HIV epidemic it is clear that wide ranging HIV interventions on a national scale is urgently needed – above and beyond the current status quo - to curb the growth of the epidemic. The proposed 90-90-90 strategy holds great promise, but in the context of the current poor viral suppression rates, there is little evidence that the final 90% object can be achieved. It is clear, that continued surveillance of the epidemic is needed to capitalize on the expansion of treatment. In the context of mass interventions, phylogenetic and phylodynamic methods, applied to the ever-increasing number of viral genotypes that are being produced, can provide insights about the underlying dynamics that is shaping the epidemic in the country. This knowledge in turn could be used by local and national health services to design better-targeted interventions in the future.

## Methods

### Ethical considerations

The use of sequences from public sequence repositories like Genbank and the HIV database at Los Alamos National Laboratory (LANL) are not subject any Ethics board as they are within the public domain. The sequences from the Division of Medical Virology, Tygerberg National Health laboratory Services (NHLS), were obtained with a waiver of informed consent from the Health Research Ethics Committee (HREC) of Stellenbosch University (IRB0005239), reference number N11/02/054. The use of sequences from the Centre for the AIDS programme of Research in South Africa (CAPRISA) and the Africa Health Research Institute (AHRI) are governed by the biomedical Research Ethics Committee of the University of KwaZulu-Natal (UKZN) and are registered under the following numbers: BREC ref number BF269/13, BREC ref number BF001/16, BREC ref number BFC189/16, HREC ref number N11/02/054. HREC and BREC comply with the South African National Health Act No 612003 and the United States code of Federal Regulations title 45 Part 46. The committees also abide by the ethical norms and principles for research as established by the Declaration of Helsinki, the South African Medical Research Council (SAMRC) Guidelines as well as the South African Department of Health (SA-NDoH) Guidelines.

### Sampling

HIV sampling was drawn from various sources, including regional surveillance sites, national drug resistance cohorts, and public domain sequences contained in databases. We chose the HIV *polymerase* (*pol*) gene for our phylogenetic reconstruction due to its relative abundance and its wide use for clinical management and surveillance of drug resistance. Sampling was drawn from four major sources including: a large national HIV drug resistance cohort managed by the Division of Medical Virology at Stellenbosch University, community surveillance platforms of CAPRISA and AHRI, and all public HIV *pol* sequences from southern African countries with known dates of isolation (position 2250–3300 relative to HXB2). All sequences were aggregated into one single data frame and curated with dates of isolation, sampling/GPS locations, gender, age and any other covariates that could be linked to genotypes.

Our dataset combined from our four different sources contained in total 15,332 sequences. The national HIV drug resistance dataset comprised the bulk of sequence (n = 5,926) followed by publicly available sequences from the Los Alamos National Laboratory HIV specific sequence database (n = 4,371; date of access 19^th^ May 2017). CAPRISA and AHRI each contributed 1,661 and 3,374 respectively to the dataset.

### Phylogenetic reconstruction and clade identification

First, all sequences were subtyped with the REGA v 3.0 subtyping tool^17^ and the stand-alone version of the jumping profile Hidden Markov Model (jpHMM) subtyping tool (http://jphmm.gobics.de)^18^. All non-subtype C isolates were removed from the dataset prior to phylogenetic reconstruction. As viral recombination can seriously affect phylogenetic and phylodynamic inference we analysed all sequences for the presence of recombination signal in RPD4^19^. RDP4 utilizes a combination of non-parametric recombination detection methods, such as RDP^20^, Bootscan^21^, Chimaera^22^, SiScan^23^, and 3Seq^24^.

Next, HIV-1 subtype C polymerase (*pol*) sequences were aligned against one another along with a homologous section of the HXB2 reference strain^25^ in Muscle v 3.5^26^. The resulting alignment was manually edited in Geneious v 8.1.8 until a perfect codon alignment was achieved and codon positions associated with major HIV drug resistance mutations were removed (https://github.com/olli0601/big.phylo). The resulting alignment was used to infer a maximum likelihood (ML) genealogy with IQ-TREE^27^. Prior to tree inference the ModelFinder package^28^ in IQ-TREE was used to identify the best fitting model of nucleotide substitution. Simultaneously, we screened for possible duplicate sequences in our sequence alignment with the help of IQ-TREE, which has a build in functionality to detect identical sequences quickly and easily. Subsequently the ML-tree topology was inferred with the general time reversible (GTR) model of nucleotide substitution^29^ and a gamma correction for among site rate variation^30^. The ultrafast bootstrap method was utilized to infer support for branching in the tree topology (1000 replicates). Clades representing putative independent introductions of HIV-1 subtype C into South were identified in the resulting ML-tree with PhyloType^31^ using the following basic search parameters: size ≥ 25 of South African sequences; difference ≤2 (which allows two or less non-South African sequences in each per clade); local branch or bootstrap support ≥95%, and an intra-cluster genetic cut-off threshold. We varied the intra-cluster genetic distance cut-off between 2% and 10% at 0.5% intervals to identify the optimal threshold for our data. We restricted the minimum size to ≥25 sequences in order to insure that a large enough sample was obtained that would allow for molecular clock analyses. We allowed a difference of <2 sequences (i.e. non-South African sequences) per clade as its clear that there are a large degree of viral flow between different southern African countries. With regards to branch support we purposefully set strict cut-offs to reduce the possibility of identifying a false clade. Finally, we choose to identify clades using PhyloType over other methods such as ClusterPicker^32^ or HIV-TRACE^33^, as this method allows us to search for phylotypes or clades that match a specific characteristic or geographic sampling location.

### Phylodynamic modelling of clades

Prior to phylodynamic and phylogeographic reconstruction, each clade was evaluated in TempEst (http://beast.community/tempest) to quantify temporal signal. All clades that had a positive correlation between genetic diversity and time were analysed to infer their estimated date of origin, as well as their temporal growth and geographic dispersal through time.

Clades were analysed in BEAST v 1.8.4 (http://beast.community/beast) under a relaxed lognormal molecular clock, an estimated mutation rate and different coalescent tree priors to infer the tMRCA and viral *Ne* dynamic through time. For each clade five different tree priors were tested: constant and exponential growth^34^, as well as non-parametric Bayesian-Skyline^35^, Bayesian-SkyRide^36^, and Bayesian-SkyGrid^37^ tree priors. Each demographic coalescent model was run in duplicate for 1 billion Markov Chain Monte Carlo (MCMC) generations, with samples being drawn from the posterior every 100,000 generations. Good mixing and convergence in the chains were assessed in Tracer (http://beast.community/tracer) based on high Effective Sample Sizes of each estimated parameter (ESS > 200) after the first 10% of the samples were discarded as burn-in. Bayes Factor comparison between different coalescent tree priors were performed to determine which coalescent model fitted the data best.

To assess the epidemic growth potential in clades, each one was also analysed under an uncorrelated log-normal relaxed molecular clock model and a Birth-Death Skyline Serial coalescent tree prior^11^. The mutation rate for the birth-death coalescent model was fixed at 1.8 × 10^−3^ mutations/site/year, based on previous estimates for HIV subtype C *pol*^6,8^. Additionally, we used the GTR substitution model with a gamma distributed rate variation across sites in BEAST v 2.4.6 (www.beast2.org). A two-dimensional sampling proportion was enforced with the sampling fixed to zero prior to the first sample date in each clade followed by an estimated sampling proportion. The MCMC was run for 1 billion generations and samples drawn from the posterior every 100,000 generations. Again, proper mixing was assessed in Tracer based on high ESS values after discarding the first 10% of posterior values as burn-in. R_0_ estimates, the becoming un-infectious rate, and the sampling proportion were plotted over time using the ggplot2 package in R^38^.

### Spatiotemporal reconstruction of South African clades

The origin and spatiotemporal dynamics of subtype C clades were investigated using a Bayesian phylogeographic approach implemented in BEAST v 1.8.4^39^. Analysis were performed under a relaxed uncorrelated lognormal molecular clock model^40^ with the GTR + G + I nucleotide substitution model. The substitution rate was modelled by a uniform distribution with mean 1.8 × 10^−3^ substitutions/site/year^6,8^ and the non-parametric Bayesian SkyRide model was used as coalescent tree prior^36^. Bayesian stochastic search variable selection (BSSVS) was employed to estimate viral migrations between locations^41^. MCMC chains were run for up to 300 million generations and proper mixing was evaluated individually in TRACER v 1.6 excluding an initial 10% for burn-in. Maximum clade credibility (MCC) trees were computed in TreeAnnotator v1.8.4, and visualized in FigTree v1.4.3. Migratory events were summarized using the SPREAD platform^42^.

## Supplementary information

Supplementary Information

## Data Availability

All genotypes that were used for epidemic dynamic reconstruction in this article have been submitted to public sequence databases. The HIV drug resistance cohort (n = 5,926) can be accesed under the following Genbank accession numbers: KX539549-KX544796. A small representative sample of AHRI sequences can be accessed from Genbank under the following accession numbers: MH920641 - MH920852. The complete AHRI dataset can be accessed from their data repository (https://www.ahri.org/ahri-publishes-updated-longitudinal-datasets/) under the following 10.23664/AC_HIVpol_full1068. Sequences from CAPRISA can be obtained through a data request from the CAPIRSA data repository (http://www.caprisa.org/Pages/CAPRISA%20Studies). All sequences that were retrieved from LANL (n = 4,371) are all in the public domain. Final alignments and the geographic co-ordinates can be obtained through personal communication with the corresponding author and is subject to the Insitutional Review Board (IRB) guidelines that govern their use.
