# Supplementary material for: The effect of interventions on the transmission and spread of HIV in South Africa: a phylodynamic analysis
Source: Sci Rep. 2019 Feb 25;9:2640. doi: 10.1038/s41598-018-37749-3 (PMC6389914; doi:10.1038/s41598-018-37749-3)
Supplement: Supplementary file 1 — Supplementary Information [file 41598_2018_37749_MOESM1_ESM.pdf]

Supplementary Materials: The effect of interventions on the transmission and spread of HIV in South Africa: a phylodynamic analysis

Eduan Wilkinson[1-3*], Dennis Maletich Junqueira[2,3], Richard Lessells, Susan Engelbrecht[4,5], Gert van Zyl, Tulio de Oliveira[2,3], Marco Salemi[1]

1. Emerging Pathogens Institute, University of Florida, Gainesville, Florida, United States of America, 32608.
2. KwaZulu-Natal Research Innovation and Sequencing Platform (KRISP), Nelson R Mandela School of Medicine, University of KwaZulu-Natal, Durban, South Africa, 4001.
3. School of Laboratory Medicine and Medical Science, Department of Health Sciences, University of KwaZulu-Natal, Durban, South Africa, 4001.
4. Division of Medical Virology, Department of Pathology, Faculty of Medicine and Health Sciences, Stellenbosch University, Tygerberg, Cape Town, South Africa.
5. National Health Laboratory Services (NHLS), Tygerberg Coastal, Cape Town, South Africa.

*Corresponding author: Eduan Wilkinson
Email: ewilkinson@ukzn.ac.za / ewilkinson83@gmail.com
Address: Nelson R Mandela School of Medicine, University of KwaZulu-Natal, 719 Umbilo Road, Durban, Republic of South Africa, 4001

Appendix

Supplementary Figure 1 – page 2
Supplementary Figure 2 – page 3
Supplementary Figure 3 – page 4
Supplementary Figure 4 – page 6
Supplementary Figure 5 – page 8

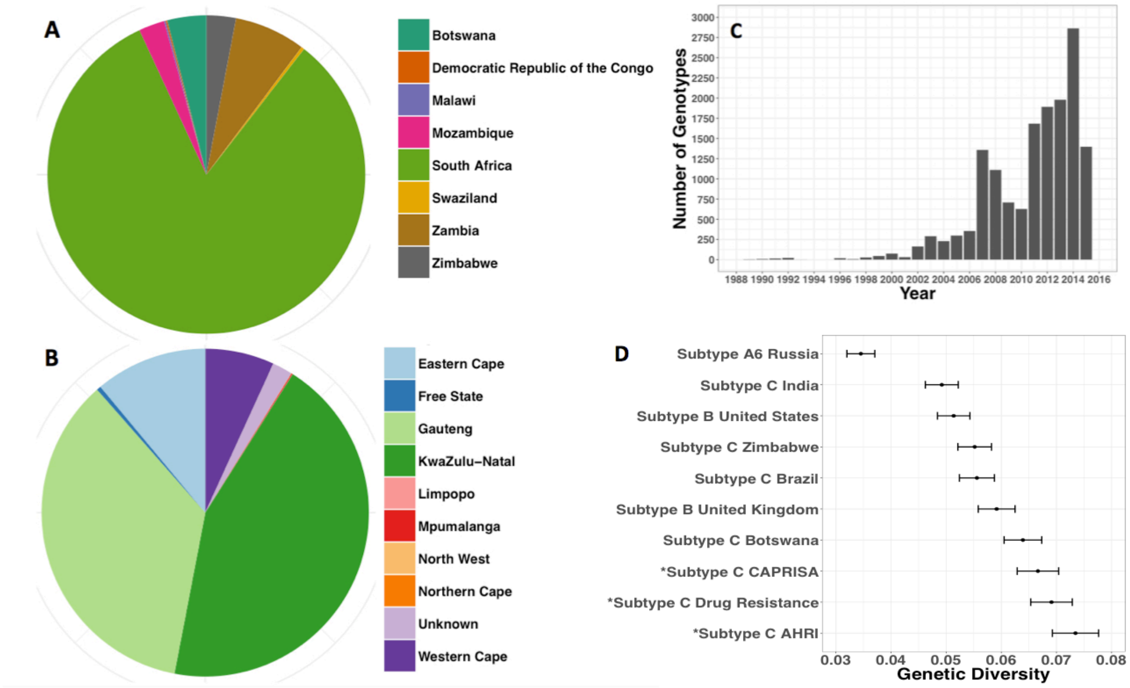

Supplementary Figure 1: Breakdown of the southern African dataset of 15,257 HIV-1 subtype C sequences. The chart in SFig1A illustrates temporal changes in sampling broken down by country of origin. The pie chart in SFig1B provides a breakdown of sequences from South Africa based on the province/state of sampling. The histogram in SFig1C provides a temporal breakdown of sampling of the entire southern African region through time. The plot in SFig1D represents the mean overall genetic diversity and the 95% confidence intervals for a random sample of 100 sequences for different geographic locations. Datasets from South Africa have been annotated with an asterisk (*).

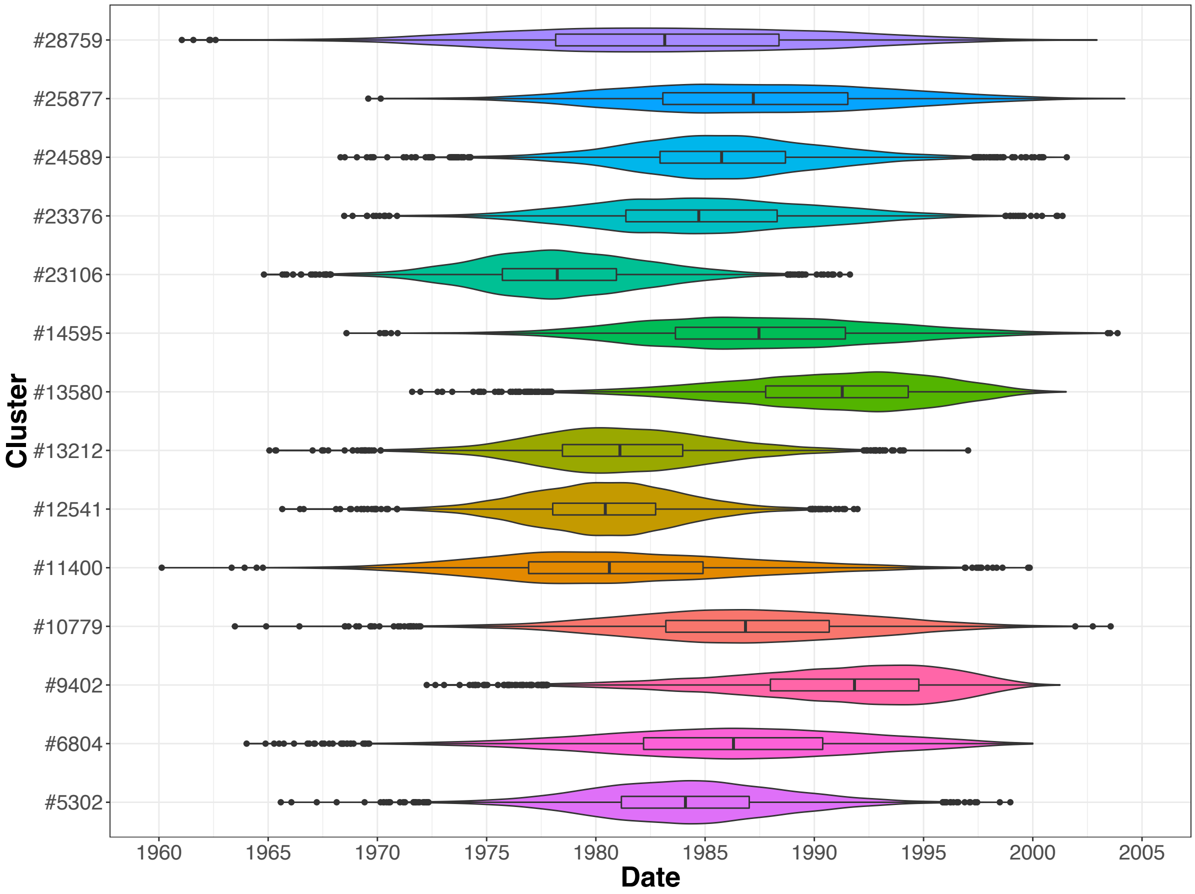

Supplementary Figure 2: The inferred estimated tree height for 14 South African clades. The violin plots represent the posterior range of estimates after the first 10% estimates were removed as burn in. The boxplots within each of the violin plots provides a summary of the posterior estimates as well as the median estimate.

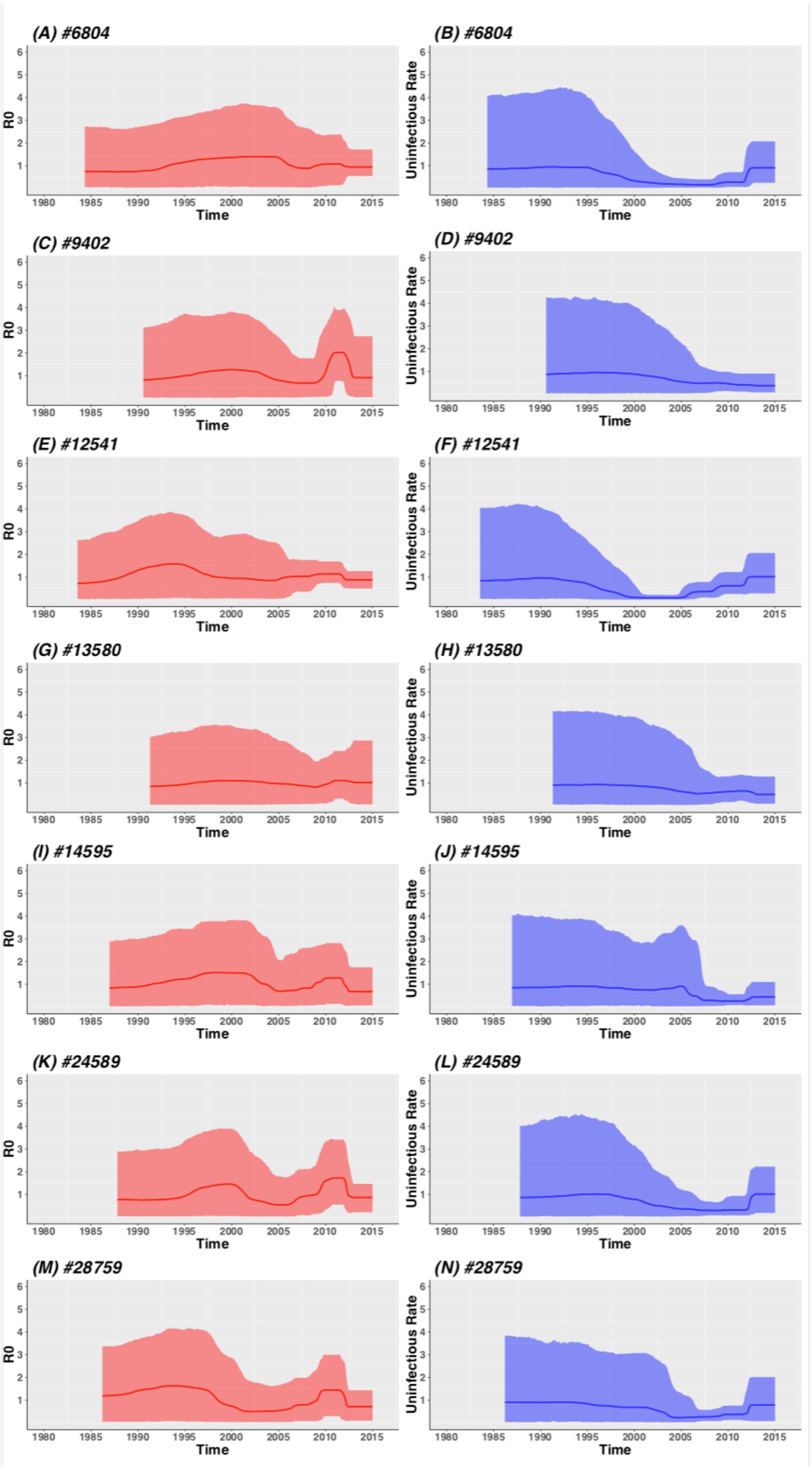

Supplementary Figure 3: The inferred temporal changes in the estimated basic reproductive number and becoming uninfectious rate for seven South African clades for whom no clear decreases in *Ne* was observed. The red solid lines represents the median estimate for R0 while the red shaded areas

represent the 95% confidence interval for R0 estimates. The solid blue lines represent the median estimate for δ, while the blue shaded areas represents the 95% confidence interval for δ estimates.

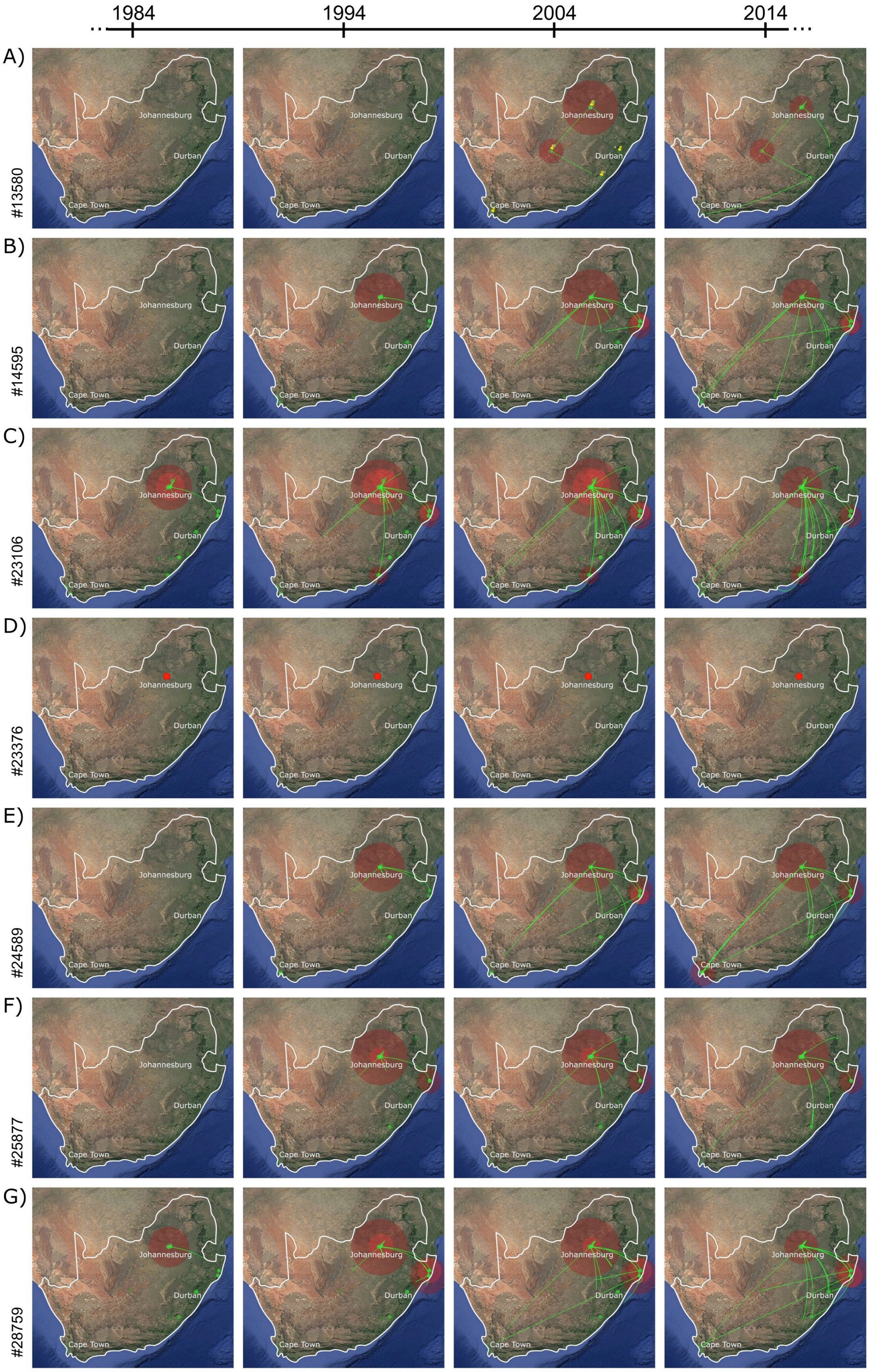

Images © 2018 Landsat / Copernicus

Supplementary Figure 4: Temporal dynamics of HIV-1 subtype C spatial diffusion in seven South African transmission clades. Lines between sites represent possible transitions of the virus between South African towns or cities. All transitions between locations have been plotted in this figure. Circle diameters are proportional to the square root of the number of MCC branches that maintain the same

location state at each time-point. The map is based on satellite pictures available in Google Earth (http://earth.google.com).

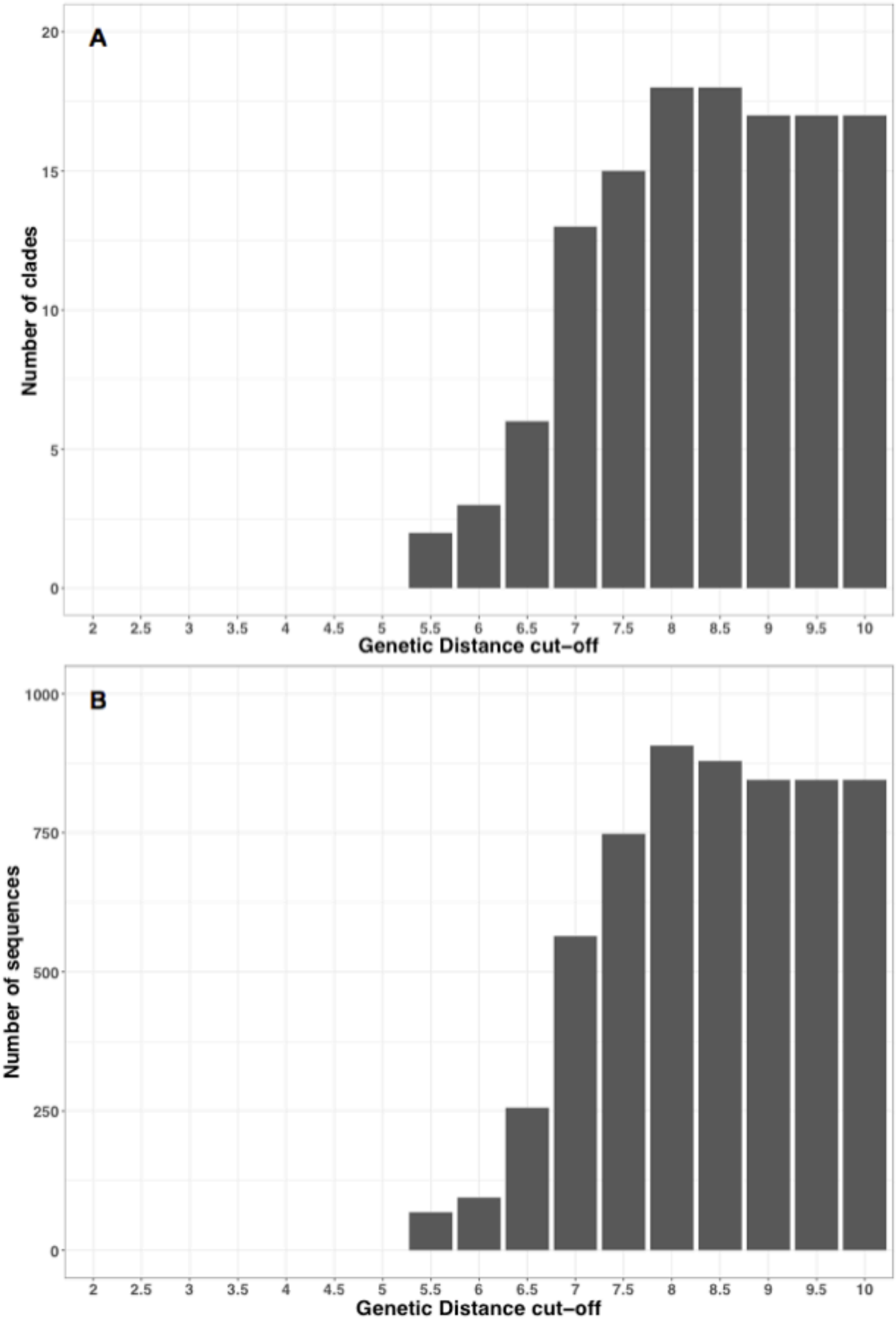

Supplementary Figure 5: Sensitivity analysis of PhyloType identification at different genetic distance thresholds. SupFig5A represents the total number of South African clades identified at various threshold cut-offs, while SubFig5B represents the total number of sequences in clades.
